# The Efficacy of Non-Pharmacological Interventions on Brain-Derived Neurotrophic Factor in Schizophrenia: A Systematic Review and Meta-Analysis

**DOI:** 10.3390/ijms17101766

**Published:** 2016-10-24

**Authors:** Kenji Sanada, Iñaki Zorrilla, Yusuke Iwata, Cristina Bermúdez-Ampudia, Ariel Graff-Guerrero, Mónica Martínez-Cengotitabengoa, Ana González-Pinto

**Affiliations:** 1 CIBERSAM, BioAraba Research Institute, OSI Araba, Department of Psychiatry, Araba University Hospital, University of the Basque Country (EHU/UPV), Olaguibel Street 29, Vitoria 01004, Spain; ksanappu@gmail.com (K.S.); inaki.zorrillamartinez@osakidetza.eus (I.Z.); cristina.bermudezampudia@osakidetza.eus (C.B.-A.); monica.martinezcengotitabengoa@osakidetza.eus (M.M.-C.); 2 Department of Psychiatry, Showa University School of Medicine, 6-11-11 Kitakarasuyama, Setagaya-ku, Tokyo 157-8577, Japan; 3 Multimodal Imaging Group-Research Imaging Centre, Centre for Addiction and Mental Health, 250 College Street, Toronto, ON M5T 1R8, Canada; yusuke.iwata2010@gmail.com (Y.I.); ariel_graff@yahoo.com.mx (A.G.-G.); 4 Department of Psychiatry, University of Toronto, Toronto, ON M5T 1R8, Canada; 5 Department of Neuropsychiatry, School of Medicine, Keio University, 35 Shinanomachi, Shinjyuku-ku, Tokyo 160-0016, Japan; 6 Geriatric Mental Health Division, Centre for Addiction and Mental Health, Toronto, ON M5T 1R8, Canada; 7 Campbell Research Institute, Centre for Addiction and Mental Health, Toronto, ON M5T 1R8, Canada; 8 Universidad Nacional de Educación a Distancia, Centro Asociado de Vitoria, Pedro de Asúa Street 2, Vitoria 01008, Spain

**Keywords:** schizophrenia, non-pharmacological interventions, brain-derived neurotrophic factor (BDNF), meta-analyses, randomized controlled trials

## Abstract

Several studies have investigated the relationship between non-pharmacological interventions (NPIs) and peripheral brain-derived neurotrophic factor (BDNF) in schizophrenia patients. We conducted a systematic review and meta-analysis to review the efficacy of NPIs on peripheral serum and plasma BDNF in subjects with schizophrenia (including schizoaffective disorder). Meta-analyses were conducted to examine the effects of NPIs on blood BDNF levels by using the standardized mean differences (SMDs) between the intervention groups and controls. In total, six randomized controlled trials with 289 participants were included. Of them, five studies used exercise, physical training or diet products. One study used cognitive training. Overall, the BDNF levels in the NPI group increased significantly compared with the control groups (SMD = 0.95, 95% confidence interval (CI) = 0.07 to 1.83, *p* = 0.03). Subgroup analyses indicated beneficial effects of a non-exercise intervention on peripheral BDNF levels (SMD = 0.41, 95% CI = 0.08 to 0.74, *p* = 0.01). Meta-regression analyses showed that the completion rate influenced the variation in SMD (*p* = 0.01). Despite insufficient evidence to draw a conclusion, our results suggest that use of NPIs as adjunctive treatments, specifically non-exercise interventions, may affect positively serum or plasma BDNF in patients with schizophrenia.

## 1. Introduction

Over the past several decades, treatments with both first- and second-generation antipsychotics (FGAs and SGAs, respectively) have certainly reduced psychotic symptoms (e.g., hallucinations and delusions) in patients with schizophrenia. However, the efficacy of FGAs and SGAs, specifically in terms of negative symptoms and cognitive impairment, has been limited. It has been suggested that a possible explanation for this lack of effect is that negative symptoms and cognitive deficits could be caused by disruptions in neurodevelopment; therefore, the modulation of a single neurotransmitter does not produce a full symptomatic response [1]. Hence, recently various types of interventions targeting inflammation and oxidative stress in particular have been used. Some examples applied in schizophrenia include the add-on treatment of omega 3 fatty acids [2], the add-on treatment of *N*-acetyl cysteine (NAC) [3], folate plus vitamin B12 [4], and mindfulness-based interventions (MBI) [5].

Brain-derived neurotrophic factor (BDNF), the most abundant of the growth factor family, has been shown to play important roles in neurodevelopment, survival and the differentiation of neurons, and synaptic plasticity [6]. BDNF protein is present in human platelets, and platelet BDNF is released into serum but not into plasma when the blood sample is processed for analysis. Peripheral levels of BDNF in serum are much higher than in plasma [7]. A considerable number of studies, at both the preclinical and clinical levels, have examined the relationship between BDNF and mental disorders, not just psychotic disorders but depression [8,9], anxiety disorder [8], post-traumatic stress disorder (PTSD) [10], and Alzheimer’s disease [11]. A recent meta-analysis demonstrated that peripheral levels of serum and plasma BDNF were moderately decreased in schizophrenia compared with controls [12]. In addition, a clinical study observed a positive correlation between plasma and cerebrospinal fluid (CSF) BDNF levels in drug-naïve first-episode psychotic (FEP) subjects [13]. With respect to the association between antipsychotic therapy and changes in BDNF levels in schizophrenia, findings from previous studies are inconsistent, although the same meta-analysis [12] demonstrated that the plasma levels of BDNF, not serum levels, increased with antipsychotic treatment independent of each patient’s response to medication.

On the other hand, several trials assessing the alterations in peripheral BDNF after non-pharmacological interventions (NPIs) in schizophrenia have been conducted. For example, Kuo et al. [14] reported that a 10-week non-pharmacological weight reduction program in the day-care unit, (including lifestyle modification, psychosocial/behavioral therapy, and exercise), reduced body weight with an elevation in serum BDNF levels in schizophrenia. In addition, Shiina et al. [15] noted that 8-week augmentation therapy with sulforaphane (SFN)-rich broccoli sprout extract improved some domains of cognitive function in schizophrenia, whereas there was no remarkable change in serum BDNF after the trial. Thus, the aim of the current study was to review the efficacy of NPIs on peripheral serum and plasma BDNF in subjects with schizophrenia.

## 2. Methods

### 2.1. Search Strategy and Selection Criteria

In accordance with the Preferred Reporting Items for Systematic Reviews and Meta-Analyses (PRISMA) [16] guidelines and the recommendations of the Cochrane Collaboration [17], we conducted a systematic computerized literature search of EMBASE, MEDLINE, and PsycINFO from inception until 5 November 2015 with no restrictions on language in order to avoid language publication bias. The following search terms were used: (schizophreni*) AND (brain derived neurotrophic factor OR bdnf). The reference lists of the identified original articles and reviews were also hand searched for additional studies that may have been missed. The study eligibility criteria are shown in Table 1. We made a decision regarding whether to include articles based on these criteria.

The protocol was registered with PROSPERO, registration number CRD42015029701 [18].

### 2.2. Data Extraction

Two authors (Kenji Sanada and Iñaki Zorrilla) independently screened all titles and abstracts to identify possible articles for full text retrieval. The full texts of potentially eligible studies were assessed independently by the same two reviewers (Kenji Sanada and Iñaki Zorrilla). Any discrepancies or divergences were resolved through discussion and consensus, and when in doubt, the final decision was made in consultation with a third author (Ana González-Pinto). When the necessary data were not available from the included studies, we contacted each author and requested the information.

We extracted the following variables: characteristics of the participants (i.e., number of participants, diagnoses of participants, diagnostic procedure, conditions of participants, age, gender, completion rate, duration of illness, daily dose of antipsychotics in chlorpromazine equivalents, body mass index, and smoking status), characteristics of NPIs and controls (contents and duration of intervention), type of control group (i.e., active control, AC, or passive control, PC), BDNF levels, BDNF measurement and assessments (i.e., source, unit, kit, collection time and total time points), and study design (study locations and sources of funding).

### 2.3. Assessment of Risk of Bias

Risk of bias was assessed with the Cochrane Collaboration’s tool [19] to assess possible sources of bias: sequence generation, allocation concealment, blinding of participants and personnel, blinding of outcome assessment, incomplete outcome data, selective outcome reporting, and other sources of bias. An assessment of the validity of the included studies was carried out by two reviewers (Kenji Sanada and Iñaki Zorrilla) independently, and any divergences were resolved through discussion or consultation with a third reviewer (Ana González-Pinto).

### 2.4. Data Synthesis

Review Manager (RevMan) version 5.3 (http://tech.cochrane.org/revman, The Cochrane Collaboration, London, UK) was used to conduct the meta-analysis and the subgroup and sensitivity analyses. Standardized mean differences (SMDs) between the intervention and control groups were determined by calculating the differences in the BDNF levels between the mean changes, namely the differences between pre- and post-intervention scores. The inverse variance statistical method and random effects model were used to adjust for study heterogeneity [20]. In cases where there were two control groups (i.e., AC and PC) in a study, we used PC and calculated the SMD between the groups. The 95% confidence interval (95% CI) was also calculated [21,22]. Effects were categorized as small (SMD = 0.2), indicating a small difference in BDNF levels between the intervention and control groups, moderate (SMD = 0.5), and large (SMD = 0.8) [23].

We tested the heterogeneity using the *I*^2^ statistic, assuming a value of 25% to indicate low heterogeneity, 50%, moderate, and 75%, high [24,25,26]. We also calculated the *Q* statistic and the associated *p*-value. A significant *p*-value (<0.05) indicates the presence of heterogeneity. Because the analyses showed that the studies were heterogeneous, leave-one-out sensitivity analyses [26] were performed to assess the potential influences of any one single study on the pooled SMD and associated *p*-values.

Subgroup analyses were conducted with the mixed effects model to evaluate possible differences according to the intervention (exercise group or other groups) and comparison groups (AC or PC).

The direction of the effects was positive if patients presented increased BDNF levels and negative if they showed decreased BDNF levels after intervention within the group.

A comprehensive meta-analysis (www.meta-analysis.com) was used to perform meta-regression analyses, taking separately the mean age, percentage of male patients, completion rate, duration of intervention, daily dose of antipsychotics in chlorpromazine (CPZ) equivalents, and body mass index (BMI).

Publication bias was assessed initially through the construction of a funnel plot analysis [27,28,29]. Egger’s test was used to contrast the null hypothesis with biased absences [30], and Duval and Tweedie’s trim and fill procedure [27] provided a number of studies that were probably absent.

All of the tests were bilateral and they performed with a significance level of *p*-value <0.05, except for the bias-related tests, which were unilateral.

## 3. Results

**Figure 1:**
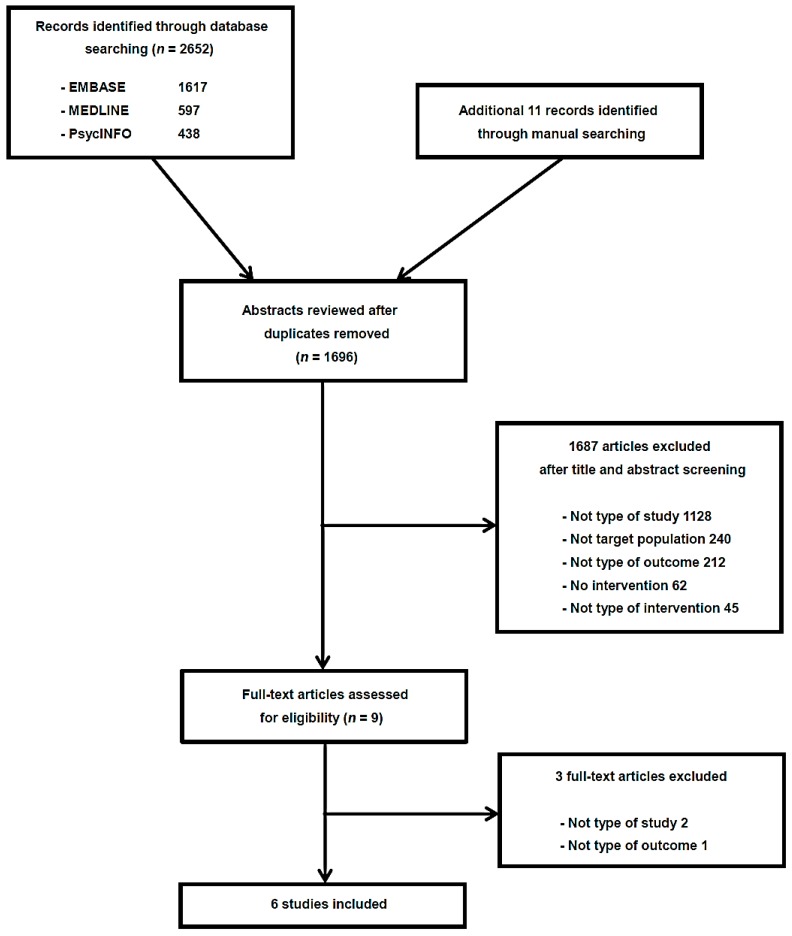
Flow diagram of study selection.

The PRISMA flow diagram of the study search and selection process is displayed in Figure 1. We included six randomized controlled trials (RCTs) with 289 participants (273 cases with patients and 16 healthy controls) in total.

### 3.1. Included Randomized Controlled Trials (RCTs)

The characteristics of each included study are summarized in Table 2. The studies were published from 2009 to 2015 and conducted in North America (*n* = 3) [31,32,33], East Asia (*n* = 2) [34,35], and the Middle East (*n* = 1) [36]. Of the included six RCTs, only two studies [31,34] were performed under AC conditions and one [31] was under a two-arm control design, i.e., AC and PC. The sample size ranged from 33 to 72 (mean: 48.1 ± 14.9). The mean age of subjects varied from 32.3 ± 10.2 to 53.5 ± 9.9 years. The proportion of males was 68.9% ± 5.7%. Only two studies reported that the length of illness ranged from 9.3 ± 7.3 to 25.3 ± 9.6 years [34,36]. The mean antipsychotic daily dose in CPZ equivalent doses [37,38,39,40] varied from 258.9 ± 232.5 to 733.5 ± 432.5 mg.

Three categories of NPIs were used in the included studies: exercise or physical training, diet products, and cognitive training. Almost all studies except one [31] were carried out using exercise or physical training [32,34,35] or diet products [33,36]. One study [31] used cognitive training. The average duration of each intervention was 10.6 ± 2.4 weeks (from 8 to 14).

All but one study [33] measured BDNF levels using an Enzyme-Linked Immunosorbent Assay (ELISA) kit (Table S1). BDNF measurements were primarily taken at two time points: before and after the intervention. One study measured the levels of BDNF not only pre- and post-intervention but also at another time during the intervention [31]. BDNF was assessed in serum, except for in one study [34] that measured plasma levels. Two trials [32,34] collected BDNF in the morning and another one [31] in the early afternoon, while the other three [33,35,36] did not note the collection time.

### 3.2. Risk of Bias

The risk of bias in the included studies is shown in Figures S1 and S2. Of the included six RCTs, five studies (83.3%) were judged as having an “unclear risk” for performance bias because they did not clarify the methods for blinding participants and personnel. The allocation concealment and blinding of the outcome assessment were also undeclared, leading to “unclear risk” in three studies (50.0%) for the detection bias, respectively. Four studies (66.6%) were regarded as at “high risk” of attrition bias due to an inappropriate application of simple imputation. Four studies (66.6%) did not report all the data of the study’s pre-specified outcomes and were considered “high risk” for reporting bias. Regarding other sources of bias, one study (16.6%) did not reveal funding support and it was judged as having an “unclear risk”, although the other studies were supported by government or non-profit organizations. Finally, only one study (16.6%) was considered to have a “low risk” for bias, being of high quality.

### 3.3. Meta-Analyses

**Figure 2:**
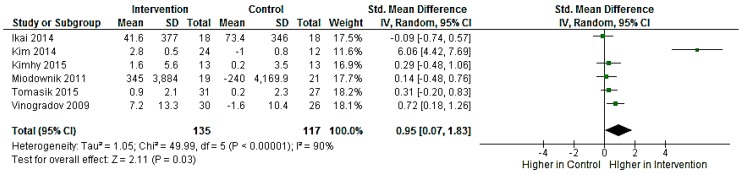
Forest plot graph of effects of non-pharmacological interventions (NPIs) on peripheral BDNF levels. There were significant effects of NPIs in peripheral BDNF levels in the intervention groups compared with controls. BDNF, brain-derived neurotrophic factor; CI, confidence interval; IV, inverse variance; SD, standard deviation; Std, standard.

In the NPI group, the overall analysis showed a significant increase in peripheral BDNF levels compared with controls (SMD = 0.95, 95% CI = 0.07 to 1.83, *p* = 0.03) as shown in Figure 2. As the studies were highly heterogeneous (*I*^2^ = 90.0%, *p* < 0.001), sensitivity analyses were conducted to determine whether an individual study was responsible for this heterogeneity. The results remained highly significant in each case (*I*^2^: from 91.0% to 92.0%), except when one single study [35] was excluded (*I*^2^ = 0.0%). In addition, we exploratory examined the effects of one outlier study [35] on BDNF levels. The results showed significant effects of NPIs on BDNF when the study was excluded (SMD = 0.31, 95% CI = 0.04 to 0.58, *p* = 0.02).

### 3.4. Subgroup Analyses

The results are shown in Figures S3 and S4. We conducted the subgroup analysis by dividing the studies according to the intervention (exercise or non-exercise) and type of control (AC or PC). In the studies with exercise intervention [32,34,35], the beneficial effects of NPIs on BDNF levels were not found. On the other hand, we found that the studies with non-exercise interventions [31,33,36] showed increased BDNF levels (SMD = 0.41, 95% CI = 0.08 to 0.74, *p* = 0.01). In the subgroup analysis of the control groups (AC or PC), no significant effects were observed in either group. In all subgroup analyses, the heterogeneity remained high, except for the subgroup of non-exercise, in which it was significantly decreased (*I*^2^ = 5.0%).

### 3.5. Meta-Regression Analyses

**Figure 3:**
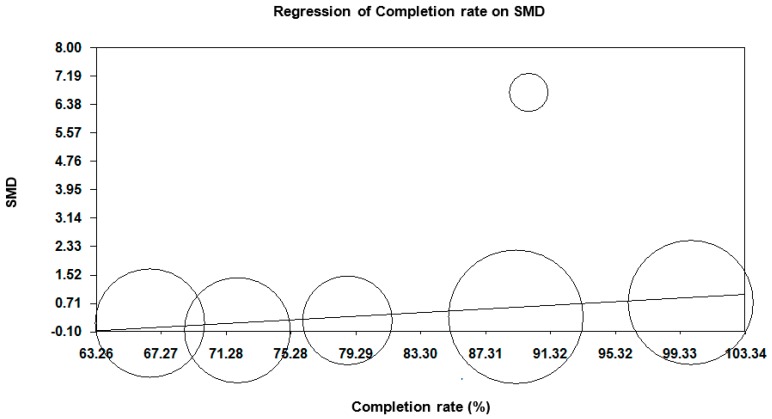
Meta-regression of the effects of completion rate in percentage on SMD. Completion rate had a positive association with SMDs of effects of NPIs on blood BDNF levels (slope = 0.025, 95% CI = 0.004 to 0.047, *p* = 0.019). BDNF, brain-derived neurotrophic factor; CI, confidence interval; NPIs, non-pharmacological interventions; SMD, standardized mean difference.

We found there was a significant association between the SMDs of the effects of NPIs on peripheral BDNF levels and the completion rate (Figure 3). This association indicated that the lower the completion rate, the lower the SMDs of the effects of NPIs on blood BDNF levels (slope = 0.025, 95% CI = 0.004 to 0.047, *p* = 0.019). There were no associations between the SMDs and age, proportion of males, duration of intervention, antipsychotic dose in CPZ equivalent dose, and BMI (Figure S5).

### 3.6. Publication Bias

The publication bias statistic of Egger’s test was significant (*p* = 0.04). The SMDs were not changed when the trim-and-fill method was used (SMD = 1.00 to 1.00). The funnel plot is shown in Figure S6.

## 4. Discussion

Despite a host of existing trials addressing the effects of pharmacological interventions (PIs) on peripheral BDNF levels in schizophrenia, while discrepant findings have been observed both in serum and plasma levels of BDNF [41,42,43,44,45,46,47,48], few studies have examined the association between NPIs and peripheral BDNF levels in patients with schizophrenia, and the results of studies have been discordant. This meta-analysis included six RCTs with 289 participants in total.

To the best of our knowledge, this is the first meta-analysis to examine the effects of NPIs on blood BDNF levels in schizophrenia. Our findings demonstrate that BDNF levels increase significantly with NPIs compared with other control treatments. This increase is consistent with the recent meta-analysis regarding the influence of PIs on peripheral BDNF levels, although the increase is only in the plasma levels of BDNF, not the serum levels, after PIs were found [11].

In subgroup analyses, we demonstrated significant effects of non-exercise interventions [31,33,36], including diet products and cognitive training, on blood BDNF levels in schizophrenia. With respect to these non-exercise interventions, few studies have reported the relationship between these types of interventions and BDNF levels in schizophrenia. For example, aside from these three interventions, 8-week sulforaphane (SFN) supplementation therapy was found to improve some domains of cognitive function in schizophrenia, while no significant change in serum BDNF levels was found [14]. Other studies only using animal experiments reported that the levels of BDNF in rodents were increased in response to a spatial learning task [49] or cognitively stimulating environments [50]. Meanwhile, findings from previous studies have noted that a decreased expression of BDNF may be related to schizophrenia patients. For example, a reduction in BDNF concentrations has been reported in the prefrontal cortex (PFC) and hippocampus in schizophrenia compared with controls [51,52,53,54]. A reduction in peripheral BDNF levels has been reported in patients with schizophrenia compared to healthy controls [31,41,44,55,56], including medication naïve and first-episode schizophrenia patients [12,43,57,58,59,60]. However, a few studies reported increased serum BDNF levels [61,62] or no significant differences in serum BDNF levels [63,64]. In addition, note that peripheral levels of BDNF are significantly correlated with cerebrospinal fluid (CSF) concentrations [12,65,66].

To the contrary, no significant effects were found in the subgroup analysis with respect to exercise interventions, while one outlier study [35] showed a large effect of combined exercise on serum BDNF. It is not clear whether this finding is specific to chronic patients with schizophrenia, is due to hospitalized patients, or is applicable to patients with first-episode schizophrenia. Exercise or physical training, however, is one activity used to increase the levels of BDNF in the brain, as already noted [67,68,69]. Moreover, although the findings did not show a pronounced effect on plasma BDNF, some previous RCTs demonstrated the benefits of yoga therapy in schizophrenia compared with exercise training, specifically in negative symptoms and social function [70,71,72]. A recent RCT also concluded that both aerobic exercise and yoga groups for early psychosis improved neurocognitive function [73]. Several factors may cause differences in the results of this analysis, e.g., age, completion rate, exercise intensity, and duration of intervention. Given that exercise and yoga therapy have a positive effect in schizophrenia, further trials with exercise training including yoga under conditions with moderate intensity and duration for schizophrenia patients could be designed to determine their effect on BDNF levels.

In meta-regression analyses, we demonstrated that the completion rate was associated with the effects of NPIs on BDNF levels, while no correlations were found between the effects of BDNF and age, proportion of males, duration of intervention, antipsychotic dose in CPZ equivalent dose, and BMI. However, the association between age and BDNF levels in this population has been reported. For example, a meta-analysis identified the evidence of a positive correlation between lowered levels of peripheral BDNF in schizophrenia and increasing age [74]. Another meta-analysis also showed evidence of an inverse relation between lowered blood BDNF levels and a greater illness length [11]. Future studies are needed to investigate the relationships between alterations in peripheral BDNF and some variables, including age, proportion of males, completion rate, duration of intervention, antipsychotic doses, and BMI.

As a whole, the findings indicate the possibility that NPIs may have a favorable effect on blood BDNF in schizophrenia patients, although the efficacy of each NPI was unclear. In addition, it is not yet known whether similar alterations in peripheral BDNF that are observed in other psychotic diseases can be improved by NPIs. Moreover, increases in BDNF caused by NPIs can be studied in a healthy population. Hence, further research is needed to clarify the effects of NPIs on blood BDNF in healthy subjects, as well as in the patients with psychotic diseases, including schizophrenia.

Our study has several limitations. Firstly, the number of RCTs addressing NPIs on peripheral BDNF in schizophrenia is limited. Only six RCTs were included in this review. Moreover, their quality was evidently low, as only one study was considered high quality [31]. Our results need to be interpreted with caution; Secondly, the number of subjects in each study was small, as the mean sample size was 48.1 ± 14.9; Third, most participants were men (the percentage of men: 68.9% ± 5.7%). This gender imbalance may lead to bias in the findings; Fourthly, no study has evaluated the long-term effects of NPIs, that is, no included trials incorporated a follow-up period. Future research protocols concerning NPI implementation with a focus on alterations in blood BDNF levels in schizophrenia may find it beneficial to include a follow-up period; Fifthly, a high level of heterogeneity was found among NPIs. They can be divided into three categories: exercise or physical training, diet products, and cognitive training; Sixthly, a high diversity among the control groups, i.e., AC and PC, was found; Finally, of the included trials, only two studies reported smoking status in each subject [31,32]. Pre-clinical and clinical studies have noted the relationship between nicotine and alterations in BDNF expression [75,76,77,78]. For future research, more attention might be paid to the influence of smoking on BDNF levels.

## 5. Conclusions

The results of our review show that, while the efficacy of each NPI on serum or plasma BDNF levels in patients with schizophrenia was inconsistent, NPIs have a beneficial effect on peripheral BDNF in schizophrenia. As several unresolved questions still exist however, further studies are clearly required, with a focus on the following points: (1) NPIs, particularly non-exercise interventions, including diet products and cognitive training, may present with pronounced effects on BDNF; (2) longitudinal RCTs with a follow-up duration in a healthy population, as well as in patients with a psychotic disease, may contribute to elucidate the specific features in each NPI; and (3) the characteristics of patients with schizophrenia (i.e., age, gender ratio, daily antipsychotic doses, BMI, and smoking status) and NPIs (i.e., duration of intervention) may affect the levels of blood BDNF after NPI.

## Figures and Tables

**Table 1:** Study eligibility criteria.

Inclusion Criteria		Exclusion Criteria
Participants	Schizophrenia and/or related disorders, or these patients and a healthy population; No restrictions were placed on age and the diagnostic procedures	Patients with other disorders, only healthy subjects
Interventions	Non-pharmacological interventions (NPIs) with usual antipsychotic treatment were eligible	Only pharmacological interventions
Outcome	At least circulating serum or plasma levels of BDNF outcomes	Only other biomarkers
Study design	Only randomized controlled trials (RCTs)	
Publications	Published as full-text articles in peer-reviewed scientific journals	Published as reviews, case reports, conference abstracts, or letters

Abbreviations: BDNF, brain-derived neurotrophic factor.

**Table 2:** Characteristics of included studies.

Study, Country	Intervention	Controls	Population	Mean Age (Years)	Gender (% Male)	Completion Rate (%)	Duration of Illness (Years)	CPZ Equivalent (Mean mg/day)	BMI (kg/m^2^)	Smoking (%)	Funding
Kimhy et al., 2015 [32], United States	Aerobic exercise (*N* = 16) 12 weeks	Treatment as usual (PC) (*N* = 17)	SZ or related disorders DSM-IV Outpatients	I: 36.6 ± 10.4 PC: 37.2 ± 9.9	I: 63 PC: 65	78.8	ND	I: 258.9 ± 232.5 PC: 439.7 ± 362.8	I: 31.6 ± 6.6 PC: 30.8 ± 5.5	I: 25 PC: 23	Government
Tomasik et al., 2015 [33], United States	Probiotic supplementation (*N* = 31), 14 weeks	Placebo supplementation (PC) (*N* = 27)	SZ or SZA DSM-IV Outpatients	I: 44.8 ± 11.2 PC: 48.1 ± 9.4	I: 71 PC: 59	89.2	ND	ND	ND	ND	Nonprofit organization
Ikai et al., 2014 [34], Japan	Hatha yoga (*N* = 25) 8 weeks	Daycare program (AC) (*N* = 25)	SZ or related psychotic disorders ICD-10 Outpatients	I: 53.5 ± 9.9 AC: 48.2 ± 12.3	I: 64 AC: 68	72.0	I: 25.3 ± 9.6 AC: 24.7 ± 11.1	I: 659.3 ± 386.2 AC: 733.5 ± 432.5	I: 24.6 ± 6.2 AC: 24.5 ± 3.1	ND	ND
Kim et al., 2014 [35], Korea	Combined exercise (*N* = 24) 12 weeks	Routine activities (PC) (*N* = 12)	SZ DSM-IV Inpatients (more than 3 years)	I: 48.7 ± 9.9 PC: 50.7 ± 12.0	ND	90.0	ND	ND	I: 25.0 ± 4.3 PC: 26.5 ± 4.7	ND	Government
Miodownik et al., 2011 [36], Israel	l-theanine supplementation (*N* = 19) 8 weeks	Placebo supplementation (PC) (*N* = 21)	SZ or SZA DSM-IV Inpatients and outpatients	I: 35.4 ± 11.1 PC: 32.3 ± 10.2	I: 90 PC: 67	66.6	I: 12.0 ± 9.8 PC: 9.3 ± 7.3	I: 500 ± 191 PC: 520 ± 296	I: 25.4 ± 6.0 PC: 25.6 ± 2.6	ND	Nonprofit organization
Vinogradov et al., 2009 [31], United States	Auditory training (*N* = 30) 10 weeks	Computer games (AC) (*N* = 26) Healthy (PC) (*N* = 16)	SZ DSM-IV Outpatients	I: 42.1 ± 9.4 AC: 46.0 ± 9.0 PC: 44.5 ± 11.7	I: 73 AC: 77 PC: 62	100	ND	I: 444 ± 477 AC: 515 ± 495 PC: NA	I: 29.0 ± 5.5 AC: 29.5 ± 4.5 PC: 26.9 ± 4.3	I: 50 AC: 43 PC: 50	Government

Abbreviations: AC, active controls; BMI, body mass index; CPZ, chlorpromazine; DSM-IV, Diagnostic and Statistical Manual of Mental Disorders, 4th edition; I, intervention; ICD-10, International Classification of Diseases, 10th edition; NA, not applicable; ND, not declared; PC, passive controls; SZ, schizophrenia; SZA, schizoaffective disorder.

## Supplementary Materials

Supplementary materials can be found at www.mdpi.com/1422-0067/17/10/1766/s1.
