# Supplementary material for: The Efficacy of Non-Pharmacological Interventions on Brain-Derived Neurotrophic Factor in Schizophrenia: A Systematic Review and Meta-Analysis
Source: Int J Mol Sci. 2016 Oct 24;17(10):1766. doi: 10.3390/ijms17101766 (PMC5085790; doi:10.3390/ijms17101766)
Supplement: Supplementary file 1 [file ijms-17-01766-s001.pdf]

Supplementary Materials: The Efficacy of Non-Pharmacological Interventions on Brain-Derived Neurotrophic Factor in Schizophrenia: A Systematic Review and Meta-Analysis

Kenji Sanada, Iñaki Zorrilla, Yusuke Iwata, Cristina Bermúdez-Ampudia, Ariel Graff-Guerrero, Mónica Martínez-Cengotitabengoa and Ana González-Pinto

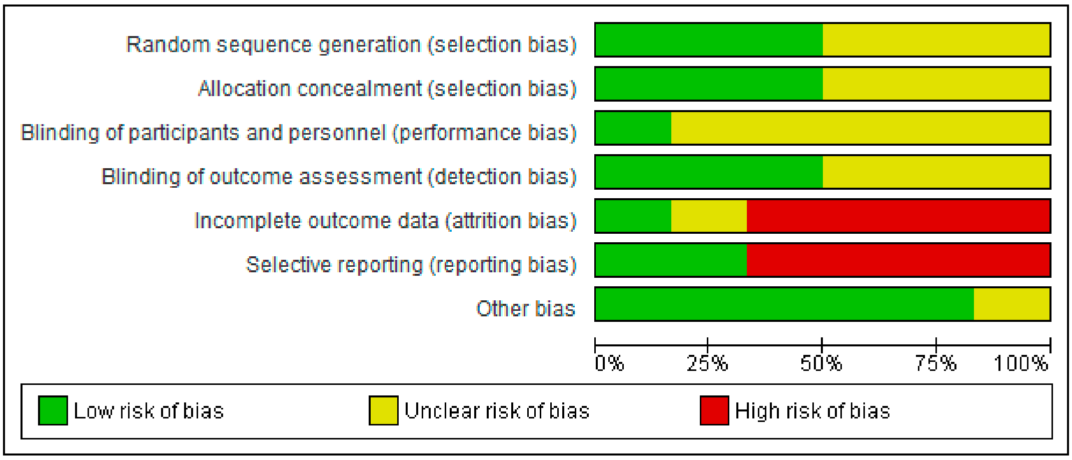

Figure S1. Risk-of-bias graph: reviews the authors' judgments about each risk-of-bias item presented as percentages across all of the included studies.

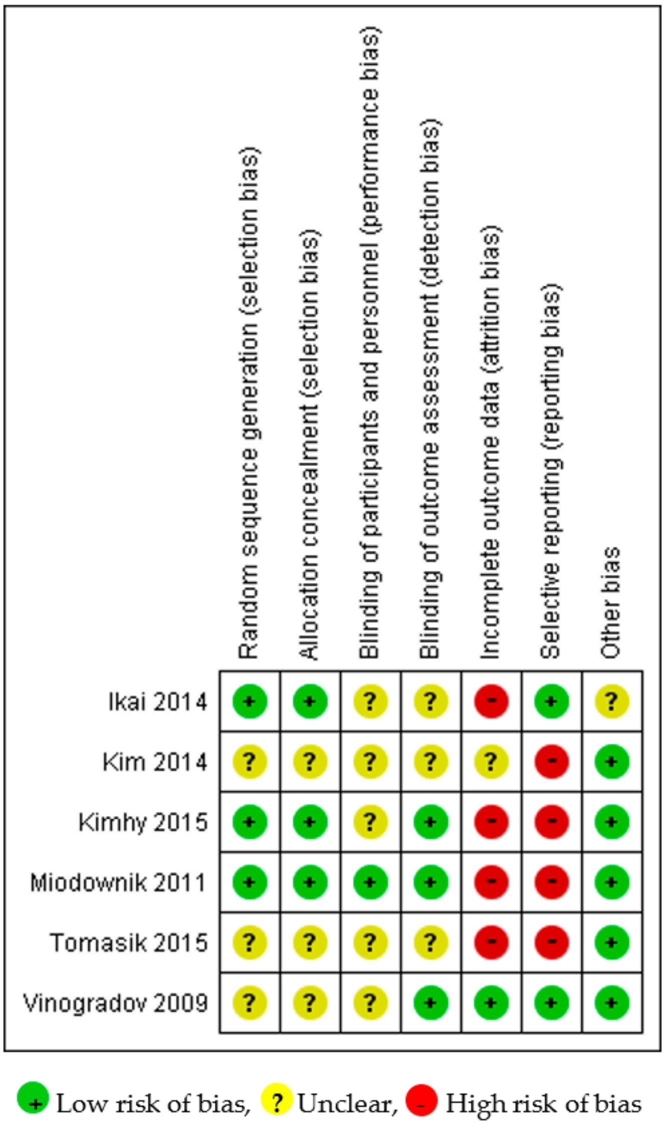

Figure S2. Risk-of-bias summary: review of the authors' judgments about each risk-of-bias item for each included study.

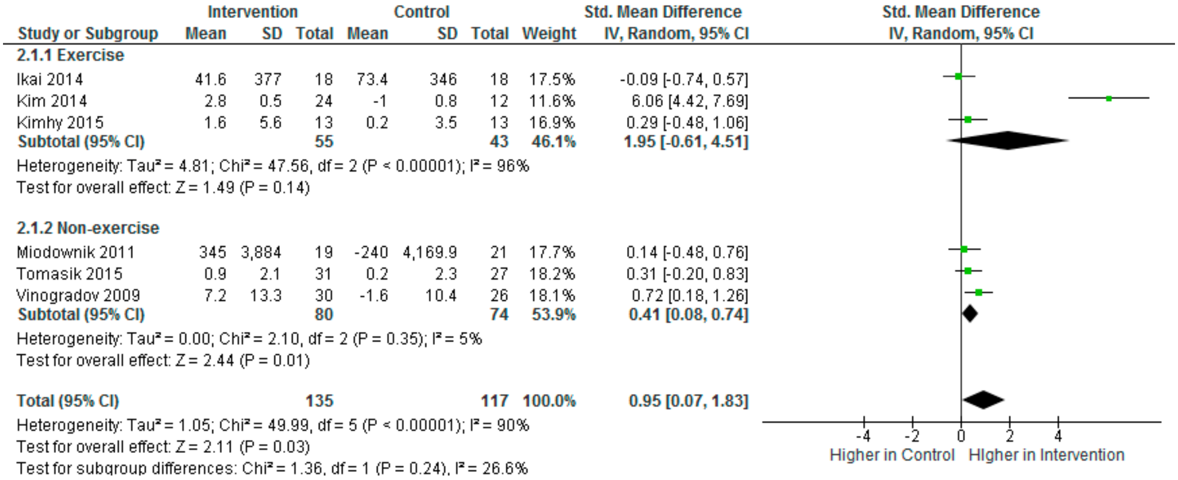

Figure S3. Subgroup analysis by type of interventions (exercise or non-exercise). Abbreviations: CI, confidence interval; IV, inverse variance; SD, standard deviation; Std, standard.

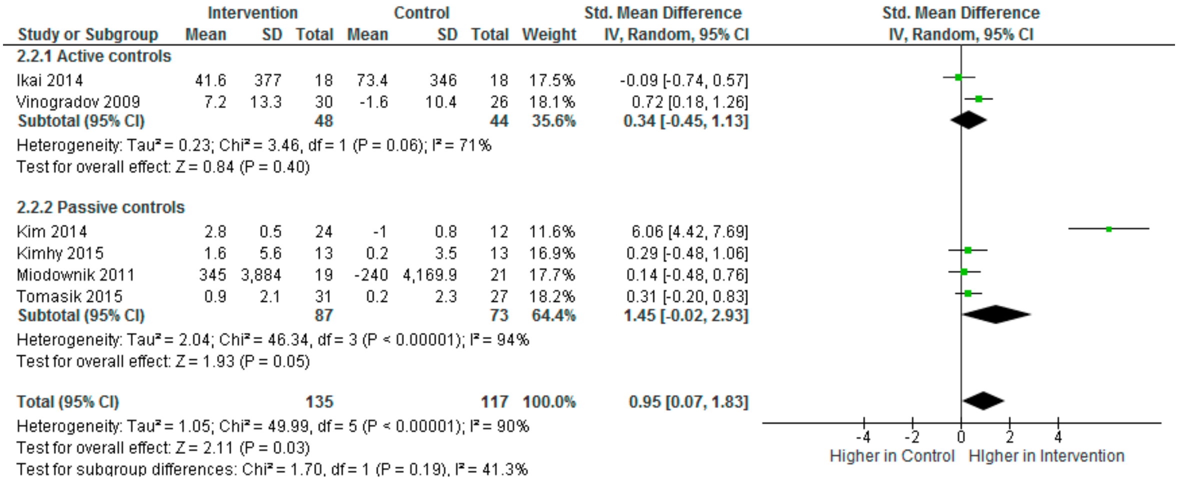

Study or Subgroup	Intervention Mean	Intervention SD	Intervention Total	Control Mean	Control SD	Control Total	Weight	Std. Mean Difference IV, Random, 95% CI
**2.2.1 Active controls**								
Ikai 2014	41.6	377	18	73.4	346	18	17.5%	-0.09 [-0.74, 0.57]
Vinogradov 2009	7.2	13.3	30	-1.6	10.4	26	18.1%	0.72 [0.18, 1.26]
**Subtotal (95% CI)**			**48**			**44**	**35.6%**	**0.34 [-0.45, 1.13]**
Heterogeneity: Tau² = 0.23; Chi² = 3.46, df = 1 (P = 0.06); I² = 71%								
Test for overall effect: Z = 0.84 (P = 0.40)								
**2.2.2 Passive controls**								
Kim 2014	2.8	0.5	24	-1	0.8	12	11.6%	6.06 [4.42, 7.69]
Kimhy 2015	1.6	5.6	13	0.2	3.5	13	16.9%	0.29 [-0.48, 1.06]
Miodownik 2011	345	3,884	19	-240	4,169.9	21	17.7%	0.14 [-0.48, 0.76]
Tomasik 2015	0.9	2.1	31	0.2	2.3	27	18.2%	0.31 [-0.20, 0.83]
**Subtotal (95% CI)**			**87**			**73**	**64.4%**	**1.45 [-0.02, 2.93]**
Heterogeneity: Tau² = 2.04; Chi² = 46.34, df = 3 (P < 0.00001); I² = 94%								
Test for overall effect: Z = 1.93 (P = 0.05)								
**Total (95% CI)**			**135**			**117**	**100.0%**	**0.95 [0.07, 1.83]**
Heterogeneity: Tau² = 1.05; Chi² = 49.99, df = 5 (P < 0.00001); I² = 90%								
Test for overall effect: Z = 2.11 (P = 0.03)								
Test for subgroup differences: Chi² = 1.70, df = 1 (P = 0.19), I² = 41.3%								

Figure S4. Subgroup analysis by control groups (AC or PC). Abbreviations: AC, active control; CI, confidence interval; IV, inverse variance; PC, passive control; SD, standard deviation; Std, standard.

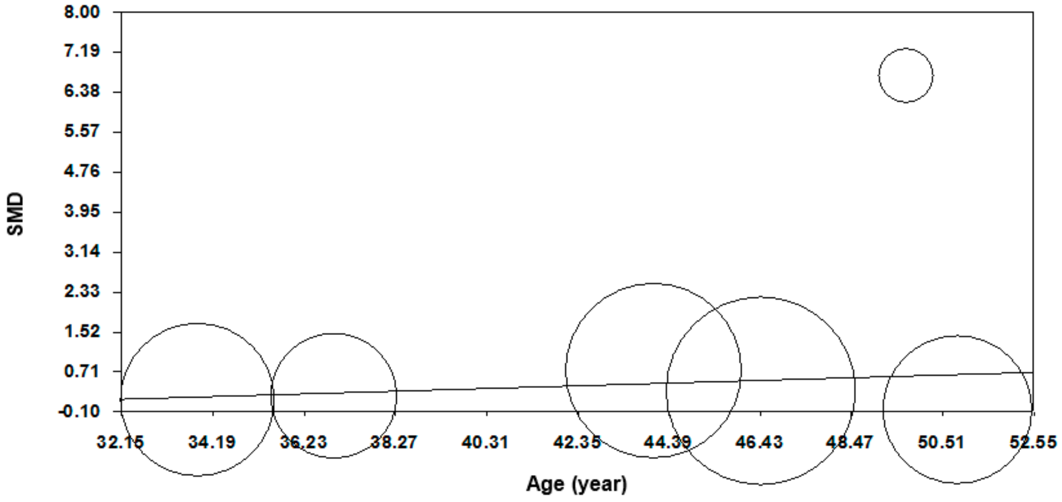

(**a**)

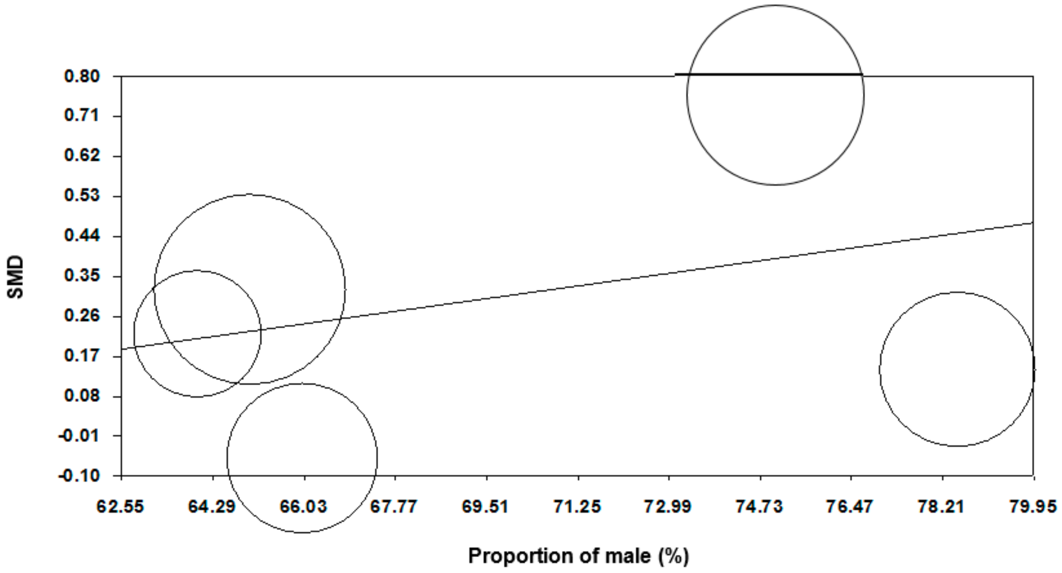

(**b**)

Figure S5. *Cont.*

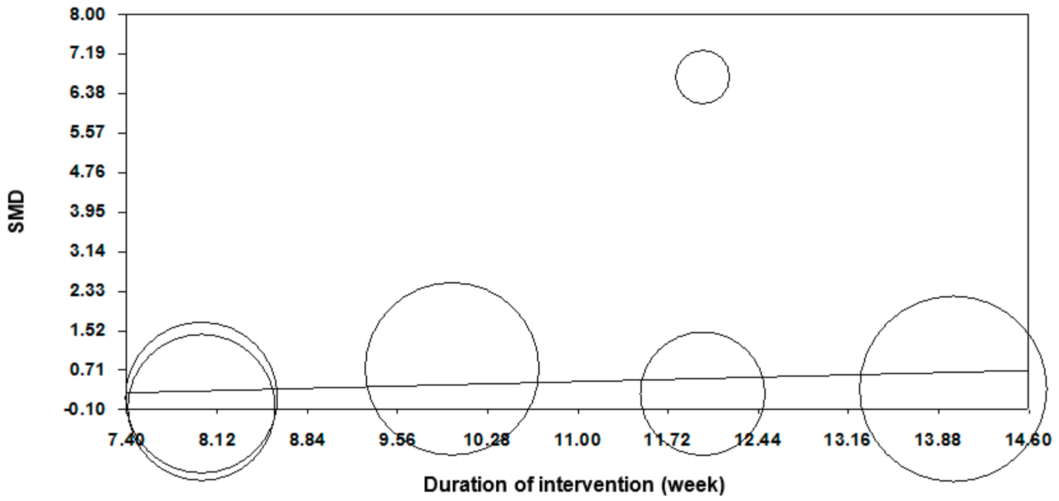

(**c**)

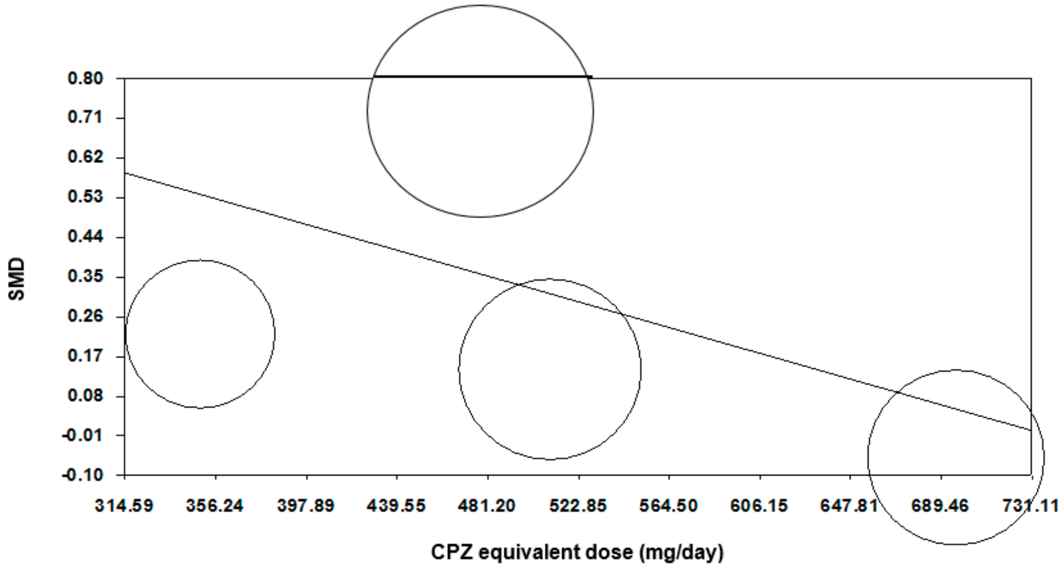

(**d**)

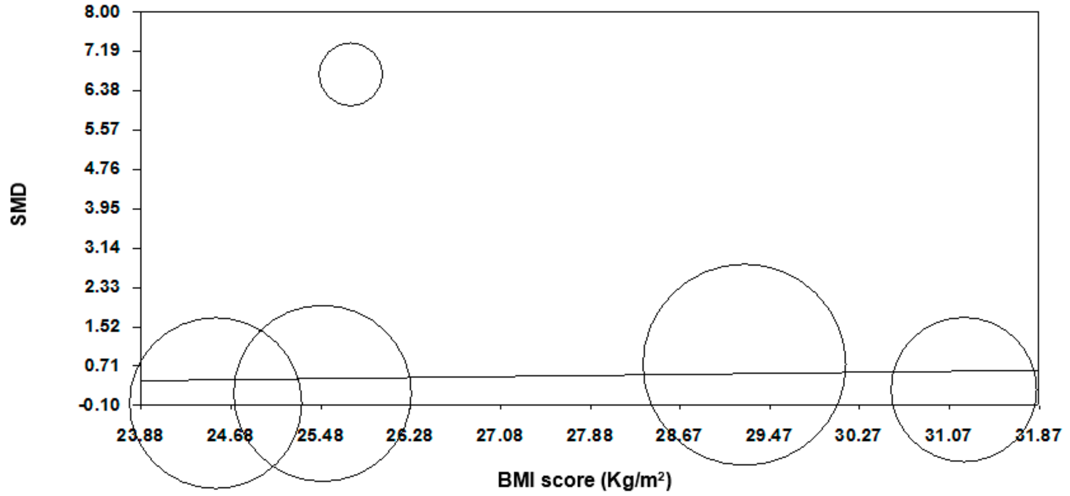

(**e**)

Figure S5. Meta-regression of moderator effects on BDNF levels by study characteristics. (**a**) Regression of Age on SMD; (**b**) Regression of Proportion of male on SMD; (**c**) Regression of Duration of intervention on SMD; (**d**) Regression of CPZ equivalent dose on SMD; (**e**) Regression of BMI on SMD. Abbreviations: BDNF, brain-derived neurotrophic factor; BMI, body mass index; CPZ, chlorpromazine; SMD, standardized mean difference.

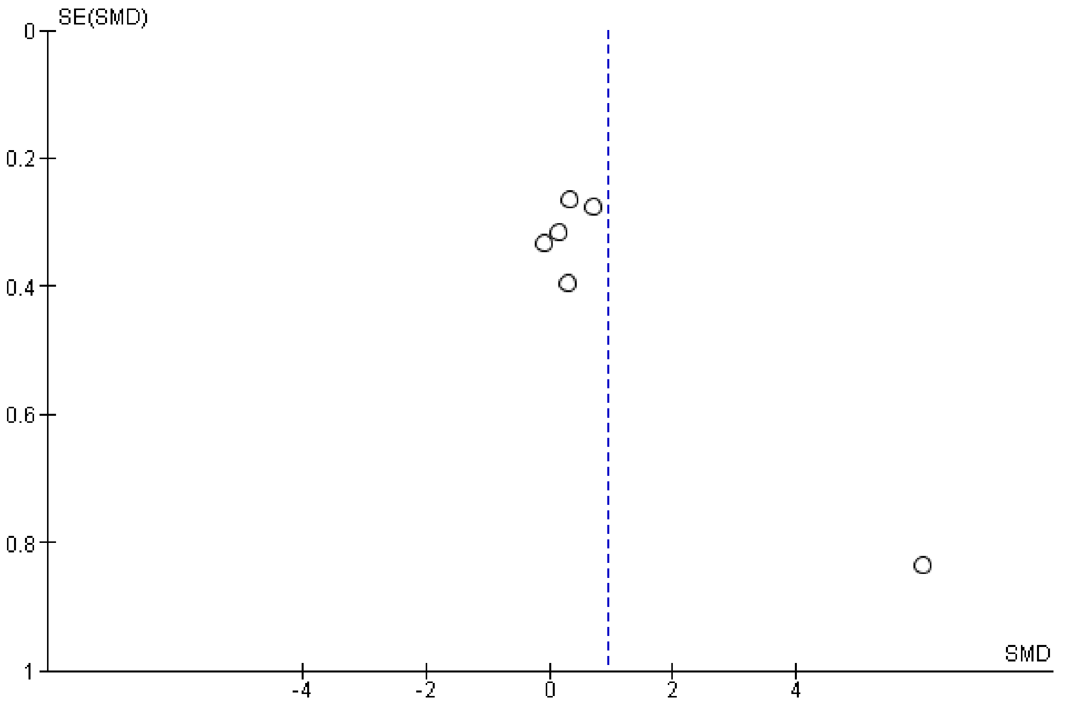

Figure S6. Funnel plot of Standard Error. Abbreviations: SE, standard error; SMD, standardized mean difference.

Table S1. Features of BDNF assessment of included studies.

Study, Country	Population	Source	Unit	Kit	BDNF	Assessments		
					Collection Time	Pre Intervention	During Intervention	Post Intervention
Kimhy et al.,2015 [32], United States	Schizophrenia or related disorders Outpatients	Serum	ng/mL	ELISA kit, R & D Systems, Minneapolis, MN, USA	~9 am Overnight fast	Baseline		Week 12
Tomasik et al., 2015 [33], United States	Schizophrenia or schizoaffective disorder Outpatients	Serum	ng/mL	Human InflammationMAP, Myriad RBM, Austin, TX, USA	ND	Baseline		Week 14
Ikai et al., 2014 [34], Japan	Schizophrenia or related psychotic disorders Outpatients	Plasma (Salivary)	pg/dL	ELISA kit, CYT306, Millipore Corp., Bedford, MA, USA	9 am~12 pm	Baseline		Week 8
Kim et al., 2014 [35] Korea	Schizophrenia Inpatients (more than 3 years)	Serum	ng/dL	ELISA kit, CUSABIO BIOTEC CO., LTD., Wuhan, China	ND	Baseline		Week 12
Miodownik et al., 2011 [36], Israel	Schizophrenia or schizoaffective disorder Inpatients and outpatients	Serum	pg/dL	ELISA kit, CYT306, Millipore product, R&D Systems, Minneapolis, MN, USA	ND	Baseline		Week 8
Vinogradov et al., 2009 [31], United States	Schizophrenia Outpatients	Serum	ng/mL	Quantikine ELISA kit, R&D Systems, Minneapolis, MN, USA	Early afternoon ~1 pm ± 1 h	Baseline	Week 2	Week 10

Abbreviations: BDNF, brain-derived neurotrophic factor; ND, not declared.
